# Retraction Note: p300 promotes proliferation, migration, and invasion via inducing epithelial-mesenchymal transition in non-small cell lung cancer cells

**DOI:** 10.1186/s12885-023-10880-9

**Published:** 2023-04-30

**Authors:** Xue Hou, Run Gong, Jianhua Zhan, Ting Zhou, Yuxiang Ma, Yuanyuan Zhao, Yaxiong Zhang, Gang Chen, Zhonghan Zhang, Shuxiang Ma, Xi Chen, Fangfang Gao, Shaodong Hong, Fan Luo, Wenfeng Fang, Yunpeng Yang, Yan Huang, Likun Chen, Haoxian Yang, Li Zhang

**Affiliations:** 1 Department of Medical Oncology, Sun Yat-Sen University Cancer Center, 651 East Dongfeng Road, 510060 Guangzhou City, Guangdong Province People’s Republic of China; 2 State Key Laboratory of Oncology in South China, Guangzhou City, Guangdong Province People’s Republic of China; 3 Collaborative Innovation Center for Cancer Medicine, Guangzhou City, Guangdong Province People’s Republic of China; 4 Department of Medical Oncology, The Fifth Affiliated Hospital of Sun Yat-Sen University, Guangdong Zhuhai City, People’s Republic of China; 5 Department of Clinical Research, Sun Yat-Sen University Cancer Center, Guangzhou City, Guangdong Province People’s Republic of China; 6 Department of Thoracic Oncology, Sun Yat-Sen University Cancer Center, 651 East Dongfeng Road, 637300 Guangzhou City, Guangdong People’s Republic of China

**Retraction Note: BMC Cancer 18, 641 (2018)**

**https://doi.org/10.1186/s12885-018-4559-3**

The Editor has retracted this article. After publication, concerns were raised about the apparent overlap between panels H1975/shNC and H1993/shNC in Fig. 2D. The authors sent raw data on request, where additional irregularities have been found, and the images for Fig. 2D were different. The authors did not provide a sufficient explanation of the irregularities. The Editor has, therefore, lost confidence in the integrity of the findings. Xue Hou, Haoxian Yang and Li Zhang agree to this retraction. The Editor has been unable to obtain the current email addresses for Run Gong, Jianhua Zhan, Ting Zhou, Yuxiang Ma, Yuanyuan Zhao, Yaxiong Zhang, Gang Chen, Zhonghan Zhang, Shuxiang Ma, Xi Chen, Fangfang Gao, Shaodong Hong, Fan Luo, Wenfeng Fang, Yunpeng Yang, Yan Huang, and Likun Chen.

